# Progress on *Azadirachta indica* Based Biopesticides in Replacing Synthetic Toxic Pesticides

**DOI:** 10.3389/fpls.2017.00610

**Published:** 2017-05-08

**Authors:** Suman Chaudhary, Rupinder K. Kanwar, Alka Sehgal, David M. Cahill, Colin J. Barrow, Rakesh Sehgal, Jagat R. Kanwar

**Affiliations:** ^1^Nanomedicine-Laboratory of Immunology and Molecular Biomedical Research, Faculty of Health, Centre for Molecular and Medical Research, Strategic Research Centre, School of Medicine, Deakin UniversityGeelong, VIC, Australia; ^2^Department of Gynecology, Government Medical College and HospitalChandigarh, India; ^3^Centre for Chemistry and Biotechnology, School of Life and Environmental Sciences, Deakin UniversityGeelong, VIC, Australia; ^4^Department of Medical Parasitology, Jawaharlal Institute of Postgraduate Medical Education and ResearchChandigarh, India

**Keywords:** azadirachtin, pesticides, biopesticide, *Azadirachta indica*, agro-medicinal components, nanocarriers, sustained delivery

## Abstract

Over the years, extensive use of commercially available synthetic pesticides against phytophagous insects has led to their bioaccumulation in the environment causing increased resistance and reduction in soil biodiversity. Further, 90% of the applied pesticides enter the various environmental resources as a result of run-off, exposing the farmers as well as consumers of the agricultural produce to severe health issues. Therefore, growing attention has been given toward the development of alternate environmentally friendly pesticides/insecticides that would aid an efficient pest management system and also prevent chronic exposures leading to diseases. One such strategy is, the use of neem plant's (Binomial name: *Azadirachta indica*) active ingredients which exhibit agro-medicinal properties conferring insecticidal as well as immunomodulatory and anti-cancer properties. The most prominent constituent of neem is azadirachtin, which has been established as a pivotal insecticidal ingredient. It acts as an antifeedant, repellent, and repugnant agent and induces sterility in insects by preventing oviposition and interrupting sperm production in males. This review discusses, key neem pesticidal components, their active functional ingredients along with recent strategies on employing nanocarriers, to provide controlled release of the active ingredients and to improve their stability and sustainability.

## Introduction

Pesticides are chemical substances used in agricultural practices to aid the production and yield by repelling, preventing, and destroying pests (Kumar et al., 2012). However, over the years, continuous application of synthetic pesticides in agriculture has caused accumulation of pesticidal residues in the environment leading to various chronic illnesses (Bag, 2000). According to a report by the United Nations Environment Programme (UNEP) and the World Health Organization (WHO), pesticides are responsible for poisoning around three million people and causing ~200,000 deaths each year, worldwide. Such cases are reported more in developing countries (95%) than in developed countries (World Health Organisation, 1990; Yadav et al., 2015). On the basis of the types of pest controlled, pesticides are divided into subcategories including insecticides, fungicides, herbicides, rodenticides, pediculicides, and biocides (Gilden et al., 2010). Most of these pesticides are stable compounds with long half-lives ranging from a few weeks to years due to their persistence in soil and water sources (Table 1), and they also enter the food chain leading to increased health risks (Pimentel et al., 1992). Pesticide exposure can occur via various means, such as inhalation of aerosols or droplets of pesticides smaller than 5 μm in diameter, which can be absorbed physiologically through the respiratory system. Dermal contact can also lead to exposure and poisoning, through the consumption of directly contaminated food or through food coming in contact with contaminated hands that can lead to pesticide poisoning (Yadav et al., 2015). Further, they can cross the placenta that can cause structural and functional defects to the fetus or in some cases death (Woodruff et al., 2008).

**Table 1:** **List of commercially available synthetic pesticides, with their toxicity and carcinogenic profiles**.

**Name**	**Side effects**	**Type of toxicity**	**Half life**	**Solubility**	**Carcinogenic properties**	**References**
Aldicarb (Insecticide)	Acutely toxic pesticide, causes excessive sweating. Salivation, vomiting, diarrhea, muscle twitching and difficulty in breathing.	Suppression of immune system, mutagenic, carcinogenic, effects on reproduction and development.	1.5 and 2 months	Highly soluble in water, also soluble in ethyl benzoate, acetone, xylene, and other organic solvents (17 mg/L of water at 25°C)	Colon cancer	Weichenthal et al., 2012
Chlorpyrifos (Insecticide)	Cholinesterase inhibition, salivation, dyspnoea, vomiting, diarrhea and exothalmia.	Nervous system damage, endocrine disruption	7–120 days	Soluble: (1.4 mg/L at 25°C)	Lung, Leukemia	Slotkin et al., 2006; Weichenthal et al., 2010
Parathion (Insecticide)	Headache, nausea, adverse effects on reproductive system	Severe poisoning can cause psychosis, unconsciousness, convulsions, cardiac arrest and coma	3–6 months	Soluble: (12.4 mg/L at 25°C)	Breast cancer	Garcia et al., 2003; Calaf and Roy, 2008
Monocrotophos	Headache, nausea, weakness, hypersalivation, blurred visions	Hazardous, accidental or intentional exposure can lead to death. Poisoning affects the central nervous system and causes loss of reflexes, involuntary muscle contractions and paralysis	7 days	Soluble: (1 kg/L, 20°C, water)	Lung cancer	Krause et al., 2013
Carbofuran (Insecticide)	Headache, nausea, sweating, chest pains, anxiety, blurred vision due to the rapid inhibition of cholinesterase activity by carbofuran	Poisoning can lead to various neurological, psychological and cognitive effects such as anxiety, depression, short-term memory loss, blurred vision	2–72 days	Slightly soluble in water (0.7 g/L of water at 25°C)	Lung cancer	Bonner et al., 2005
Endosulfan (Insecticide)	Difficulty in breathing, incoordination, vomiting, diarrhea	Chronic toxicity can lead to seizures, changes in kidney structure, blood chemistry	35–67 days	Slightly soluble (0.33 mg/l)	Breast, liver	Kumar et al., 2014
Atrazine (Herbicide)	Abdominal pain, vomiting, diarrhea, eye irritation, slowed breathing, muscle spasms, breathing difficulty	Animals with an oral dose: paralysis of limbs, respiratory distress, structural and chemical changes in lungs, liver, kidney, ovaries and growth retardation	In surface water: >200 days, Atmosphere: 14–109 days	Slightly soluble (0.030 g/liter in water at 20°C)	Colorectal cancer	Lerro et al., 2015
Paraquat (Herbicide)	Acute respiratory distress, thirst, nausea, headache, fever, muscle pain, nail damage, temporary nail loss	Leads to the production of free radicals and oxidative stress, causing cell death. Accelerates the development of Parkinson's disease. Paraquat can cross the placenta causing acute toxicity and death of the fetus	16 months to 13 years	Soluble: (700 g/L at 20°C)	Melanoma, Ovarian and Lung cancer	Park et al., 2009
Glyphosate (Herbicide)	Anorexia, vomiting, hypersalivation and diarrhea, dysphagia, gastrointestinal hemorrhage	Decreases body weight, increases incidence of cataract, lens degeneration, mutagenicity and reduces sperm count	2–197 days	Slightly soluble: (12 g/L at 25°C)	Breast cancer	Cox, 1995; Thongprakaisang et al., 2013
Carbendazim (Fungicide)	Acute toxicity is low, but direct contact can lead to discomfort in eye, skin irritant, irritation of respiratory tract, chronic bronchitis	Minor effects on cellular respiratory function, interference with the mitotic spindle proteins, no teratogenicity concern for dietary exposure	Soil: 8–32 days Water: 2–25 days	(8 mg/L water)	Prostate cancer	Tessier and Matsumura, 2001; Peyre et al., 2014
Mancozeb (Fungicide)	Cholinesterase inhibitor. Causes, headache, nausea, blurred vision, skin rash	Impairs thyroid function, and is mutagenic	Soil: 1–2 days Water: 4–8 weeks	Insoluble in water and most organic solvents	Thyroid cancer	Nordby et al., 2005

Most of the highly toxic pesticides are readily metabolized and eliminated by the body, however, acute short term exposure can lead to their accumulation. The active ingredients, carriers, solvents, and emulsifiers present in pesticides can cause severe side-effects (World Health Organisation, 1990). The severity of the effects of exposure is dependent on various factors such as the intake dose, route of exposure, pesticide absorption in the body, their accumulation efficacy and persistence. In most cases, metabolism of pesticides in the body makes them water-soluble, so that the body can readily excrete them. However, sometimes metabolism can increase the toxicity, for example the metabolism of carbosulfan and furathiocarb produces carbofuran which is more toxic than the native form of the pesticide. Furthermore, some fat-soluble substances are not metabolized by the body and get stored in the fatty tissues leading to their accumulation. They become even more concentrated while passing through the food chain (Ntow et al., 2008). Such cases cause various toxic effects including, skin sensitization, allergic reaction rashes, neurotoxicity, carcinogenic, reproductive, and endocrine defects, cataract formation and defects in the immune system (Alavanja et al., 2004; Owens et al., 2010). Among these, the carcinogenicity of pesticides have been well documented, and there are many reports that have linked synthetic pesticides to various types of cancers with exposure to various insecticides, herbicides and fungicides (Table 1).

Moreover, the use of synthetic pesticides has led to disturbances in the environment, causing pest resistance and toxicity to non-target organisms. In some cases, these synthetic pesticides have caused acute and chronic poisoning to farmworkers, applicators and even consumers, thus making it imperative to adopt alternative means. One of the significant alternative strategies is employing botanical pesticides, which is the most efficient means to replace the wide use of synthetic pesticides. Among these, plant based biopesticides using plant extracts and oils have proved to be the most efficient way of insect control. These herbal pesticides aid the agricultural yield (Table 2), as they can be used as insecticides, fumigants, manures, urea coating agent or soil conditioners. They can be used alone, or in combination with other herbs, so as to increase the insecticidal efficacy.

**Table 2:** **Common herbs with active ingredients containing insecticidal properties**.

**Herb**	**Active ingredient**	**Agricultural: Mechanism of action**	**References**
Plant essential oils: (Clove Eucalyptus Lemon grass Mentha species *Thymus vulgaris*)	Eugenol 1,8-cineole Citronellal Menthol Thymol and carvacrol	Fumigant and contact insecticidal property. It interferes with the neuromodulator: octapamine and GABA-gates chloride channels. (Volatile thus, limited persistence in field)	Koul et al., 2008
*Tanacetum parthenium* (Feverfew)	Pyrethrum	Neurotoxic: causes rapid knockdown effect, along with hyperactivity and convulsions. Pyrethrum blocks voltage-gated sodium channels in nerve axons (Half-life: 2 h)	Isman, 2006
Turmeric (*Curcuma longa*)	ar-turmerone and turmerone	Inhibitory activity on insect growth, antifeedant	Tripathi et al., 2002
*Ferula asafoetida* (Hing)	Asafoetida (oleo-gum-resin)	It acts as an insect repellent and consists of a characteristic unpleasant smell	Kavianpour et al., 2014
Henna (*Lawsonia inermis*)	Quinones (gives dying properties to henna)	Ethyl acetate and ethanol extracts of Henna exhibits an antifungal effect. Quinones are a source of free radicals, which are stable and complex irreversibly with the protein's nucleophilic amino acids and cause an inactivation of protein, thus exhibiting potential antimicrobial functions	Lee et al., 2001; Jeyaseelan et al., 2012
*Allium sativum*	Allicin (gives the pungent characteristic odor to crushed garlic)	Antifeedant, repellent, inhibitor of molting and respiration, cuticle disruption and fecundity reduction	Prowse et al., 2006
*Momordica Charantia* (Bitter Melon)	Crude leaf extract, bitter Momordin	Antifeedant	Devanand and Rani, 2008; Ling et al., 2008

**Figure 1:**
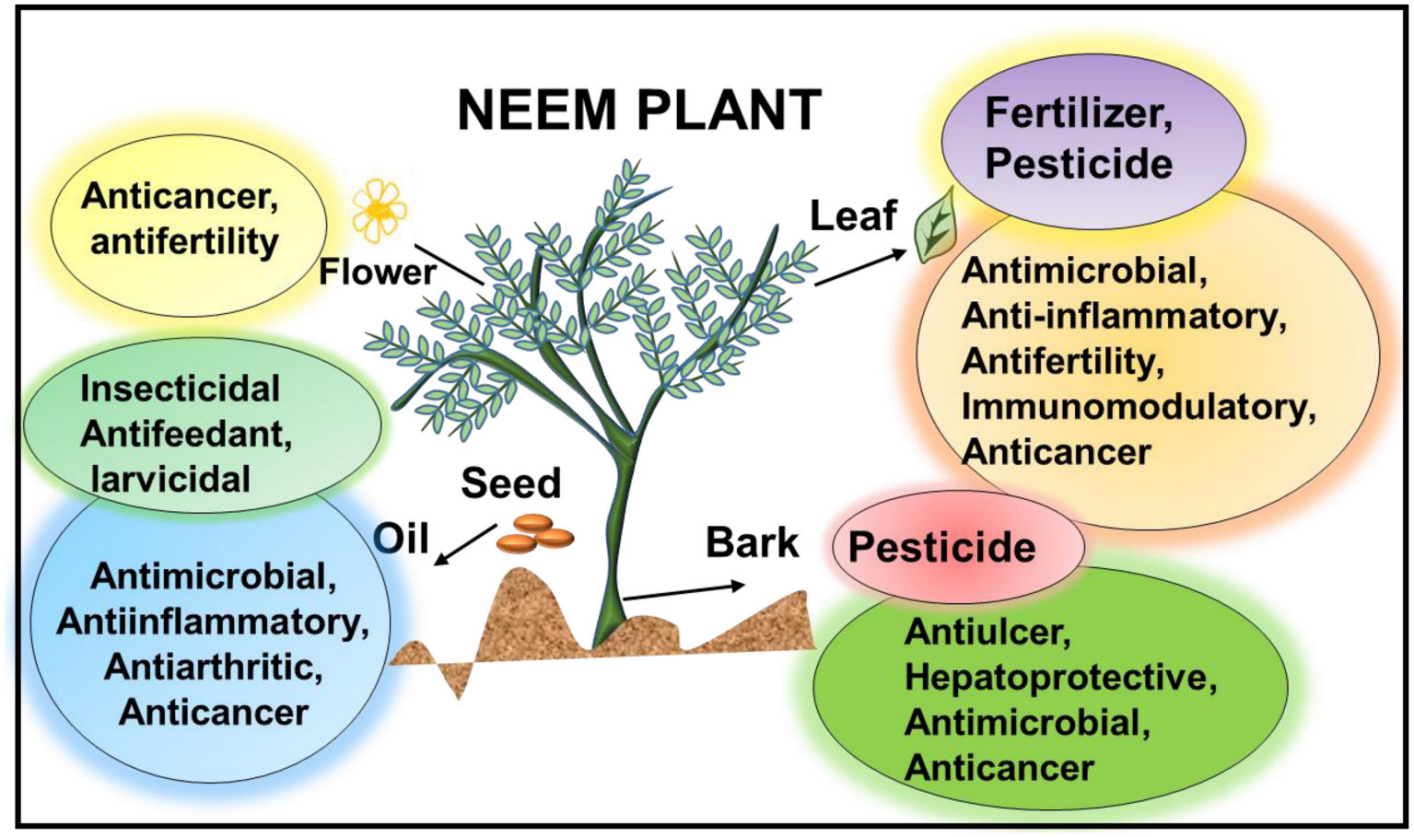
**Schematic representation of the agro-medicinal tree, ***Azadirachta indica*** indicating the potential of this tree as a biopesticidal and therapeutic agent**. Active components of neem leaf, bark, seed, and oil have anti-inflammatory, antimicrobial, anticancer, hepatoprotective, anti-arthritic, and immunomodulatory properties. Further, oil extracted from the seeds, neem bark, and neem leaf exhibits insecticidal properties and can be used as a pesticide, herbicide, fungicide and weedicide. Neem leaf can also be used as a biofertilizer as they are capable of increasing the yield of the vermicompost.

Among these herbs, Neem (*Azadirachta indica*) belonging to the Meliaceae family has emerged as a highly potent bio-pesticide (Figure 1). This evergreen, fast-growing plant known as the Indian lilac (Schmutterer, 1990) offers immense antifeedant properties due to its efficacy in suppressing the feeding sensation in insects, at concentrations even less than 1 parts per million (Isman et al., 1991). It is a draft resistant tree that thrives in a sub-humid to sub-arid climate with an annual rainfall of 400–800 mm (Schmutterer, 1990). It comprises of more than 200 allelochemicals prevalent in variable concentrations in the different parts of the plant, providing a variety of pesticidal properties (Koul and Wahab, 2004). Seeds from this tree comprises of 40% of oil with azadirachtin as the major active ingredient, that is mainly responsible for the insecticidal activity of neem (Isman et al., 1991). Further, the seed cake obtained during the processing of neem oil is a vital natural fertilizer used in the common agricultural practices. Additionally, neem leaves have been employed for centuries against the stored grain pests due to its repellent properties (Koul et al., 1990). Collectively, all parts of this plant are known to exhibit by-products that inherently impart an internal chemical defense making neem free from the pest attack, which can also be exploited to develop an efficient pest control strategy. Further, the functional ingredients of neem, exhibit, therapeutic significance as neem oil, bark, leaves and their purified biochemicals are documented to have anticancer (Paul et al., 2011) and antimicrobial (Raut et al., 2014) properties. Neem leaf extract possesses anti-inflammatory properties (Kumar et al., 2015), while neem oil acts as an antifertility agent (Kaushic, 2004). Most importantly an active ingredient of neem known as NLGP has now evolved as a potent immunomodulatory agent (Mallick et al., 2013), thus making it an ideal agro-medicinal plant (Figure 1). This unique attribute of neem makes it an ideal bio-pesticidal agent, as it does not cause non-specific toxicity to mammals.

## Bio-pesticidal activity of neem

### Neem oil

**Figure 2:**
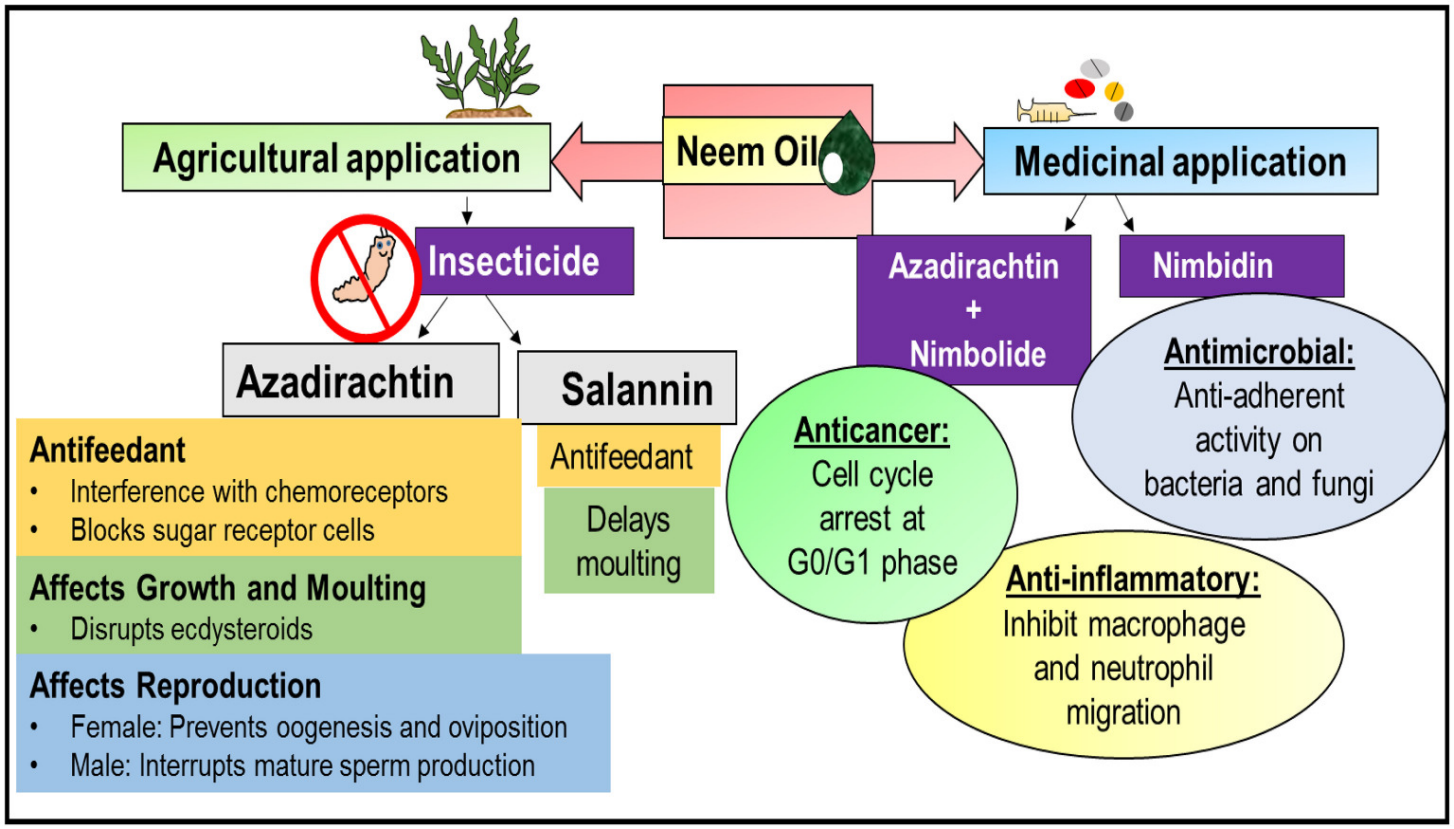
**Illustration of agro-medicinal applications of neem oil**. Azadirachtin and salannin are the major components of neem oil with insecticidal properties. They both act as antifeedants and delay the process of molting in insects. Azadirachtin and nimbolide also exhibit significant medicinal properties as they act as an anticancer agent by arresting the cell cycle. Another compound, nimbin, can also be extracted from neem oil, and demonstrates anti-inflammatory and antimicrobial properties.

Neem oil extracted by cold-pressing the seed kernels of neem is highly effective against soft-bodied insects and mites. The presence of disulphide in neem oil is a major contributor to its bioactivity. The most significant insecticidal and therapeutic properties of this agro-medicinal neem component are illustrated in Figure 2. Neem oil contains more than a dozen azadirachtin analogs, but the major contributor to the insecticidal activity is azadirachtin. The remaining triterpenoids including nimbin, salannin, and their derivatives contribute little to the efficacy (Isman, 2006). Interestingly, neem oil is non-toxic to mammals, birds and fishes and exhibits fewer chances of resistance, due to its multiple mode of action on insects. Many formulations of neem seed oil exhibit antifeedant, ovicidal, larvicidal, insect growth regulatory, and repellent activity against insect pests. The larvicidal property of neem oil against mosquitoes has long been investigated.

Mosquitoes are responsible for causing serious human diseases, that have led to millions of deaths per year, including malaria, dengue, and chikungunya. As a result, botanical origin insecticides are increasingly gaining interest, as they exhibit a multitude of components that minimize the chance of resistance to synthetic insecticides in mosquitoes. One such study investigated the potential of neem oil as an eco-friendly alternative for the control of malaria. Neem oil formulations at different concentrations were evaluated against *Aedes, Anopheles*, and *Culex* mosquitoes (Dua et al., 2009). Results indicated a decrease in the mortality rate which was 98.1% reduction in *Anopheles*, a 95.5% reduction in *Culex* and a 95.1% reduction in *Aedes* on day 1, and thereafter by day 7, 100% larval control was observed. The anti-ecdysteroidal activity observed was due to the presence of azadirachtin in neem oil that kills larvae via a growth inhibition effect. Even though the neem oil formulations employed were costlier than the synthetic larvicides, neem oil was more effective for preventing pest resistance (Dua et al., 2009). Hirose and co-workers evaluated the fungitoxic effect of neem oil, along with three other biofertilizers Supermagro, E.M-4 and Multibion^TM^ against two entomopathogenic fungi, *Metarhizium anisopliae* and *Beauveria bassiana*. The study indicated a significant negative effect of neem oil on the germination, conidial production, and vegetative growth of the two fungi, which was more significant in Multibion^TM^ (Hirose et al., 2001). The efficacy of neem oil was evaluated in a study against *Sarcoptes scabiei* var. *cuniculi* larvae, which are ectoparasites with high possibility of causing zoonotic infections. The acaricidal activity was observed to be 100% after 4.5 h of exposure to four fractions of neem oil obtained by chloroform extraction (Du et al., 2009). However, the study lacked in analyzing the long term effect of these fractions which requires special emphasis since neem oil has a low shelf life (Immaraju, 1998; Javed et al., 2007). The role of neem oil as an insect growth regulator was further evaluated by Kraiss and Cullen (2008), on a pest of soybean *Aphis glycines* Matsumura. Direct spray of two neem formulations, neem seed oil, and azadirachtin were evaluated under controlled conditions for their efficiency in deterring the fecundity, development time and survivorship of *A. glycines and its predator Harmonia axyridis*. It was observed that both neem formulations were effective in causing nymphal mortality (80% by azadirachtin and 77% by neem oil), with a significant increase in the development time of the surviving adults. However, neither of the formulations caused any significant effect on the fecundity of the insects and the mortality rate was not immediate. Further, a non-target effect of neem treatments on the larval survival and development time of *H. axyridis* was observed, which requires further investigation (Kraiss and Cullen, 2008). A recent study conducted on *Idioscopus clypealis*, a mango pest, compared the efficacy of three synthetic pesticides, endosulfan, cypermethrin, and imidacloprid, along with environmentally friendly neem oil, against the mango hopper. Although among the three insecticides tested, imidacloprid exhibited the highest efficiency against the pest. Biopesticides based on a neem oil formulation also presented significant efficacy. Therefore, the sole dependency synthetic pesticides can be easily modified by inculcating an eco-friendly management program through incorporation of neem oil for controlling the mango hopper (Adnan et al., 2014). Along with growth deterring properties, neem oil also significantly delays reproduction in pests. It causes lethal toxicity during the pupal stage leading to various morphological deformations such as malformed adults, partial ecdysis, and molt blocking, that defers and inhibits adult formation (Boulahbel et al., 2015). However, recently it has been reported that neem oil along with its pest deterrent attributes, also causes malformations in the growth and survival of a non-target predator, *Podisus nigrispinus* which is a zoophytophagous pest commonly used in the biological control of pests. An increasing morphological deformation in the wings, legs, and scutellum along with mortality was observed with increasing concentration of neem oil. Thus, it is imperative to consider, effect of neem based pesticides on, non-target predators (Zanuncio et al., 2016).

Seed cake that is obtained during the processing of neem oil can be used as a bio-fertilizer, as it provides nutrients to the plant. It performs a dual function, as a pesticide and as a fertilizer. The seed cake produces high-quality natural manure, since neem cake compounds increase nitrogen and phosphorous content in soil which, also increases the soil fertility. Powdered seed granules are used as soil conditioners to improve the quality of soil enhances plant growth (Lokanadhan et al., 2012).

### Neem leaf

Neem leaf is a source of vermicompost with fertilizer and pesticidal properties. Adding neem leaves while vermicomposting with earthworms facilitates faster growth and reproduction of earthworms in neem-fed vermireactors. They are capable of converting 7% of the feed into vermicompost per day thus, increasing the yield (Gajalakshmi and Abbasi, 2004). However, while using neem-fed vermireactors it is important to consider the powerful nematicidal potential of neem, which can have a detrimental effect on annelids (Akhtar, 2000). It increases the shelf life of mungbean grain by providing protective efficacy against *Callosobruchus chinensis*, pulse beetle. A neem leaf dose of 1.5 mg/100 g seed presents a significant decrease in the number of eggs laid as well as it increased the mortality in adults by 62% suggesting its potential as a bioactive anti-repellent during post-harvest grains/seeds storage (Ahmad et al., 2015). Recently, the antifeedant and repellent efficacy of neem leaves was validated in a study where enrichment of organic fertilizers with neem leaf powder and boiler ash was observed to significantly improve resistance of plants against infestation by aphids (Brotodjojo and Arbiwati, 2016).

### Neem bark

The use of neem bark as a bio-insecticide is limited, as its pesticidal efficacy is lower than the other components of the neem tree including neem seed and leaves in controlling insect pests (Sirohi and Tandon, 2014). However, it is known to possess phytotoxic properties when enriched in soil to control pest which was documented in a study, where neem bark and leaves inhibited germination and growth of various crops such as alfalfa, carrot, bean, rice, radish, and sesame along with various weeds thus, demonstrating allelopathic properties (Xuan et al., 2004). Neem bark extract based dyed fabric was recently shown to also exhibit anti-lepidopteran efficacy which was more significant in comparison to neem leaf extracts due to the presence of higher azadirachtin, cyanogenic glucosides, and nimbin content (Ahmad et al., 2015). Therapeutically, this component of neem tree is known to exhibit anti-ulcer and anti-secretory properties that are used to control gastric hypersecretion and gastroduodenal ulcers (Bandyopadhyay et al., 2004).

## Neem active pesticidal components

Neem parts constituting leaf, seed, bark, flower, and oil possess a multiplicity of components that are responsible for its multiple pesticidal activities.

### Azadirachtin

The main component of neem oil, leaves, flowers, and fruits with insecticidal properties is Azadirachtin. It constitutes 0.1–0.3 % of neem seeds and was first isolated from *A. indica* by Morgan et al. at Keele University, England (Schmutterer, 1985). It is a complex tetranortriterpenoid limonoid with repellent and pesticidal properties. Biosynthesis of triterpenoids from *A. indica* initiates with azadirone and a C-ring opening, which culminates in Azadirachtin formation. Azadirachtin, along with other related triterpenoids such as Azadirachtin B, salannin and nimbin, are the active ingredients in neem plant based bioinsecticides and they act by disrupting the growth and development of insects and by deterring their feeding. It is considered as a botanical pesticide with exceptional growth regulating and biocidal efficacy along with deterrent effects on the ovipositing and feeding of insects (Morgan, 2009). An attempt to evaluate the exact molecular mechanism of insecticidal activity of azadirachtin on *Monochamus alternatus*, a pine sawyer beetle, has indicated enrichment of differentially expressed genes (DEGs) in 50 pathways. 920 and 9984 unique genes were found to be up and down regulated significantly. Such detailed gene profiling to assess the azadirachtin internalization with *M. alternatus*, can promote the development of efficient azadirachtin derived herbal pesticides (Lin et al., 2016).

#### Mechanism of action

Azadirachtin is structurally similar to the insect hormones known as “ecdysones” which are responsible for metamorphosis in insects. The feeding behavior in insects is dependent on the neural inputs received from the chemical sensors of the insects, for example, the taste receptors in the mouthparts, tarsi and oral cavity. These sensors integrate a “sensory code” that is delivered to the central nervous system. Manifestation of antifeedancy by azadirachtin occurs through the stimulation of deterrent cells in these chemoreceptors and by blocking the feeding stimulation in insects by firing the “sugar” receptor cells (Jennifer Mordue Luntz et al., 1998).

In addition to antifeedancy, azadirachtin injection also leads to physiological effects in the insect's midgut, which causes a reduction in the post-ingestive digestive efficiency. This reduction in efficiency is known as “secondary” antifeedancy and is due to disturbances in the hormonal as well as physiological systems. These disturbances include hindrance in the food movement through the insect's midgut and inhibition in production of digestive enzymes (Schmutterer, 1985). An early study conducted by Nisbet et al. (1996) highlighted this antifeedant feature of azadirachtin. It was established that a concentration of 50–100 ppm of azadirachtin caused an insecticidal effect however, it has a high potential to harm beneficial insects as well. Therefore, a low concentration was tested which concluded that a concentration of only 5 ppm of azadirachtin can dramatically decrease the fecundity in aphids within 48 h of feeding. Further, a diet containing more than 10 ppm azadirachtin led to the production of non-viable nymphs. Thus, it can be concluded that even with a low concentration of azadirachtin, it cannot cause an immediate antifeedancy. Secondary antifeedancy effect as well as a sterilant effect can rapidly manifest themselves and aid in providing crop protection by reducing the pest population without harming non-target or natural predator populations (Nisbet et al., 1996).

Azadirachtin interferes with the growth and molting process of insects. Its ingestion leads to abnormal molts, growth reduction and increased mortalities. Azadirachtin interferes with the synthesis of an “ecdysteroid” hormone, which is responsible for the molting in insects. Indirectly, azadirachtin affects the neurosecretory system in insects by blocking the release of morphogenetic peptide hormones such as prothoracicotropic hormones that control the prothoracic glands and allatostatins, which in turn control the corpora allata (responsible for secreting juvenile hormones). Molting hormones from prothoracic glands are responsible for controlling the formation of new cuticle, and play a central role in ecdysis. The formation of juvenile stages during each molt is controlled by the juvenile hormone from the corpora allata (Nisbet, 2000). Disruption in these events by azadirachtin, leads to various sterility and molting defects. Moreover, cellular uptake of azadirachtin inhibits both cell division as well as protein synthesis thus, causing midgut cell necrosis and flaccid paralysis of muscles (Nisbet, 2000). Neem products influence fecundity in female insects in a dose-dependent manner. Azadirachtin prevents oviposition by inhibiting oogenesis and synthesis of ovarian ecdysteroid. In males, azadirachtin acts by interrupting the meiotic process responsible for sperm production (Linton et al., 1997).

### Nimbolide

Two main active ingredients; Nimbolide B and Nimbic acid B also demonstrate herbicidal activity of neem. Their allelopathic and phytotoxic activity was observed in a study where they inhibited the growth of lettuce, crabgrass, alfalfa, jungle rice, and barnyard grass. The allelopathic activity increased with an increase in the concentration of active compounds, but the intensity varied with different species of weed (Kato-Noguchi et al., 2014).

### Salannin

Salannin is an active component of neem with insect-growth regulating and antifeedancy activity. Salannin deters feeding, increases the larval stage duration and causes delayed molt, leading to decreased pupal weight that results in larval and pupal mortality. This has been demonstrated in an early study on *Oxya fuscovittata* where salannin caused delayed molting and nymphal mortality (Govindachari et al., 1996). The bioactivity observed was more prominent in azadirachtin as compared to salannin, however, a combination of azadirachtin with salannin and nimbin can provide insect growth-regulating activity with increased efficacy. Biological activity of salannin has also been assessed in the tobacco armyworm *Spodoptera litura* and gram pod borer *Helicoverpa armigera*. All three components of salannin, including salannol, salannin, and 3-O-acetyl salannol, exhibited strong antifeedant activity. Nutritional assays were performed to analyse the antifeedant feature of the component and a significant reduction in the growth and development of larvae fed with the neem compounds was observed, indicating feeding deterrence in insects (Koul et al., 2004). This study also supported the use of multiple active components, as they augmented the bioactivity and confirmed that the formulations had a variety of growth inhibitory, antifeedant, and toxic effects. Contrary to this, a recent study was conducted, that attempted to increase the potent variability of salannin as an insecticide molecule. The objective was to convert salannin into two metabolites N-(2-hydroxyethyl)-a,b-unsaturated-g-lactam salanninactam and g-hydroxybutenolide salanninolideb. This conversion was enabled by transforming the C-7 furan moiety using a fungal strain *Cunninghamella echinulate* (Haldar et al., 2014). This transformation of complex natural molecules into novel metabolites can be exploited for potential benefits as it can limit the use of multiple compounds in insecticidal formulations. However, the detailed mechanisms of insecticidal action of these transformed salannins are still not known.

## Procedures for the extraction of neem functional ingredients

**Figure 3:**
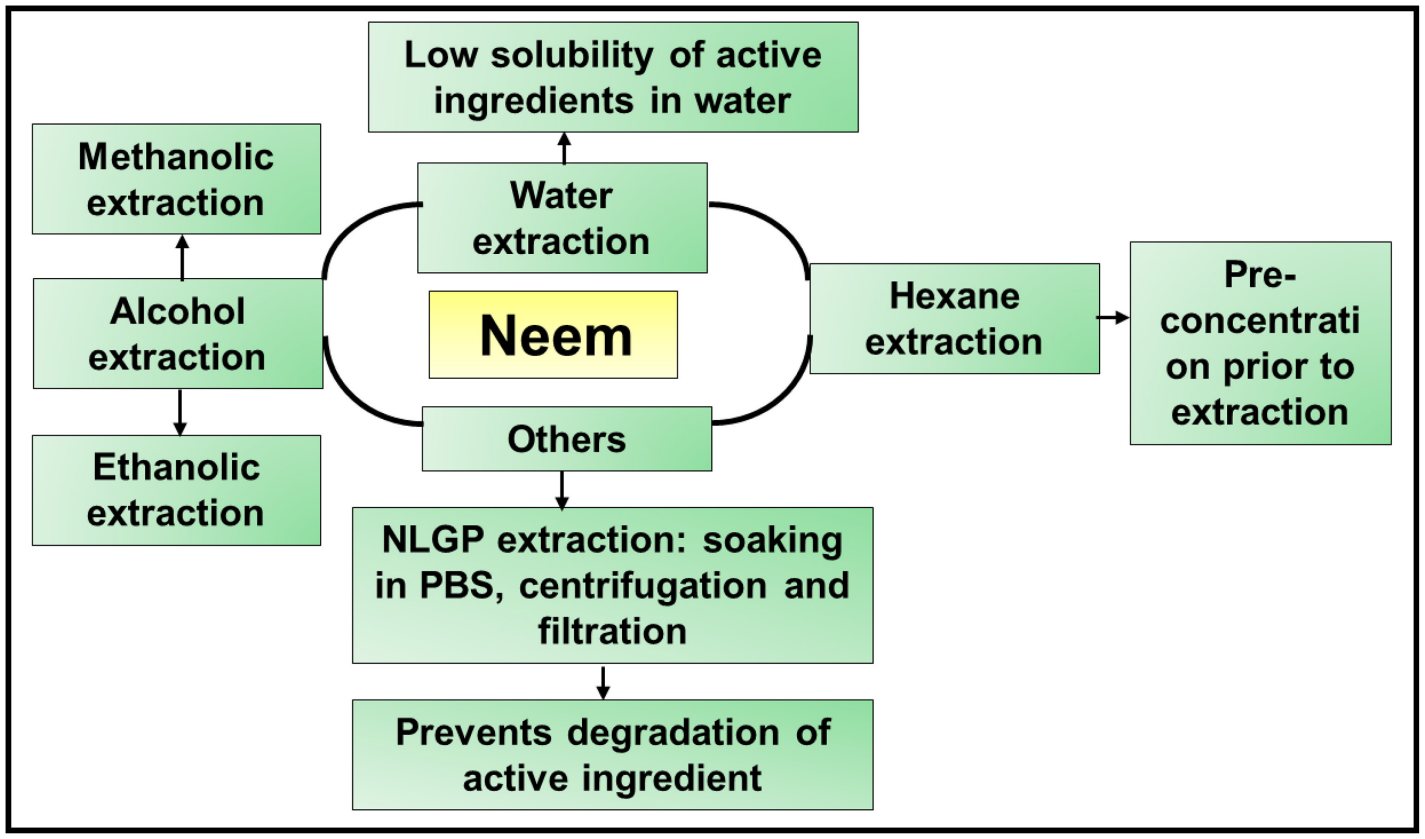
**Schematic representation of various extraction procedures used to extract the active ingredients from neem leaf, bark, and seeds**. Three major extraction procedures are employed to extract various active components of neem: water, alcohol, and hexane extractions. These techniques are used either alone or in combination to yield the limonoids. However, extraction of NLGP from neem leaves involves a different procedure, which includes soaking the neem leaf powder overnight, followed by a series of centrifugation steps and finally filtration to yield the purified protein.

All parts of the neem tree contain bioactive compounds, however the active ingredients of neem either have low solubility in water but complete solubility in organic solvents such as alcohols, hydrocarbons, ethers, or ketones, or are highly concentrated. Thus, they need to be extracted, which can be undertaken using the following methods (Figure 3).

### Water extraction

One of the simplest techniques of extraction is through crushing and grinding of kernels, followed by subsequent extraction with water. Neem seeds are usually kept overnight, and the crude suspension obtained is then filtered and used as a sprayable emulsion. However, since the active ingredients have less solubility in water, the process requires a large amount of water. One study evaluated the antifecundity effect using aqueous extracts of neem seed kernels on the development of *Bactrocera dorsalis* and *Bactrocera cucurditaei* when fed as a water source, which was then compared with pure azadirachtin. Crude extract was prepared by grinding the neem seed kernels into a fine powder, followed by subsequent extraction with distilled water using a laboratory blender. The crude extract was then filtered and dried. Results indicated that when compared to aqueous neem seed extract, pure azadirachtin had a greater effect on fertility and fecundity. The post-embryonic effect of aqueous neem seed extract was confirmed for the first time in this study, thus identifying a cheap, safe and renewable alternative to synthetic pesticides (Singh, 2003).

### Hexane extraction

Hexane extraction involves grating and steeping the neem seed kernels in the solvent hexane, resulting in the removal of oil. This oil is useful in killing the eggs of many insects such as the larvae of mosquitoes and leafhoppers that are otherwise difficult to control. Purification of azadirachtin essentially involves its enrichment through solvent extraction. Briefly, the neem seed kernels are suspended in hexane with continuous stirring and are also filtered. This concentrates the neem oil containing limonoids. This same method is applied to isolate azadirachtin from neem seeds using methanol, following which it is partitioned with hexane in order to remove any other non-polar compounds present (Sinha et al., 1999). Precipitation induced by hexane is an important pre-concentration step for the extraction and separation of neem oil limonoids. Hexane is employed because it is a very non-polar solvent. When added into neem oil, it forms a new more non-polar hexane-oil phase as compared to the oil phase, thus reducing the solubility and leading to the precipitation of the more polar limonoids from the neem oil. This method results in the formation of fine powder containing azadirachtin and other components (Melwita and Ju, 2010).

### Alcohol extraction

The most direct method for the extraction of neem constituents in concentrated form, is through alcohol extraction, as limonoids have very high solubility in alcohol solvents. During the process, kernels are grated and steeped in ethanol or methanol. The yield obtained is 50 times more concentrated than the yield obtained through water extraction. In one study, methanolic neem leaf extracts were studied for their anti-inflammatory potential. A simple extraction procedure was applied to yield green crude extract. Briefly, dried neem leaves were ground and then dissolved in methanol with continuous shaking. Solvent was then evaporated to dryness, resulting in a green crude extract (Schumacher et al., 2011). Another study employed hexane and ethanol as two solvents in a 1:5 ratio for extraction from neem seeds and evaluated the effects of temperature, type of solvent and particle size on the kinetic and thermodynamic parameters of extraction. An increase in the temperature of extraction resulted in higher oil yield, but a lower oil quality. The extraction process was endothermic, spontaneous and irreversible (Liauw et al., 2008). The resulting concentrated active ingredients, obtained through the above mentioned extraction techniques, can then be modified into dust, granules, emulsifiable concentrates and wettable powders for more sophisticated use (Liauw et al., 2008).

### Extraction of neem leaf glycoprotein (NLGP)

NLGP is a component of neem with immunomodulatory properties (Baral et al., 2010). The extraction of this active ingredient was first described by Baral and Chattopadhyay (2004). Briefly, the active component of neem leaf is isolated by soaking neem leaf powder obtained by shed drying and pulverizing neem leaves, in phosphate buffered saline (PBS), followed by a series of centrifugation and filtration steps. It is a simple yet very common technique used for the extraction of NLGP, and the extract obtained can then be analyzed for its endotoxin, protein, and carbohydrate concentration (Baral and Chattopadhyay, 2004; Chakraborty et al., 2008; Goswami et al., 2014). The anti-cancer potential of neem leaf components has been the attention of numerous alternative therapies, as its multiple active ingredients offers anti-mutagenic (Arumugam et al., 2014), anti-proliferative (Sharma et al., 2014; Patel et al., 2016) and anti-inflammatory properties (Sarker et al., 2014). Baral et al. reported that NLGP prevents the growth of murine Ehrlich carcinoma (EC) as well as B16 melanoma in mice, by inducing lymphocytosis and by stimulating the increase of cluster of differentiation (CD)4^+^ and CD8^+^. Thus, it was concluded that it inhibits tumor growth through immune activation (Baral and Chattopadhyay, 2004). In line with this, it has been observed that NLGP causes activation of natural killer (NK) cells and NK-T cells and stimulates the secretion of interferon gamma (IFNγ) and Tumor necrosis factor alpha (TNFα) leading to tumor cell cytotoxicity (Haque and Baral, 2006). The analysis of mechanism involved in NLGP mediated tumor restriction revealed the secretion of IFNγ within the NLGP treated tumor microenvironment. Further, low expressions of FasR(+) cells was observed within the CD8(+)T cells. Collectively, it has been suggested that NLGP enhances the optimal functioning of T cells inhibiting the tumor growth (Barik et al., 2013). This therapy stimulates the activation of NK/NKT cells along with initiating Th1-type immune response and thus, maintains normal immune homeostasis in immunosuppressed hosts through upregulation of type 1 response (Mandal-Ghosh et al., 2007; Bose et al., 2009). It also helps in the maturation of myeloid and mouse bone marrow derived dendritic cells providing efficient antigen presentation as well as co-stimulation of effector T cells (Goswami et al., 2010) indicating it's potential as a candidate for vaccine tool toward cancer immunotherapy. Recently, it was also established that a combination of NLGP with sarcoma antigen (SarAg) vaccination demonstrates anti-tumor immunity which had high superiority as compared to the SarAg vaccination alone since, the inoculation of the vaccination presented disease free survival until 60 days (Ghosh et al., 2016).

## Neem based nano-biopesticides

In agricultural practices, herb-based insecticides have the disadvantage of getting degraded when exposed to sunlight, due to low shelf life. Moreover, the active ingredients of neem cause non-specific toxicity. Aqueous extracts of neem leaves have shown toxicity to *Oreochromis niloticus*, by causing telangectiasis, bend in secondary lamellae, pyknosis, secondary lamellae shortening, and necrosis. Therefore, it is imperative to consider eco-toxicological properties of active ingredients of bio-pesticides (Alim and Matter, 2015). To overcome this, nano-biotechnology offers great potential, as it involves the production of unique nanoformulations that have the ability to improve the physiochemical stability, degradability, and effectiveness of natural products (Perlatti et al., 2013). These nanocapsules provide slow, controlled and cyclic assembly. It facilitates sustained release of the active compounds that can be controlled at the site of action thus, minimizing non-target toxic effects. Additionally, they prevent the loss of volatile components, thus augmenting the stability of the phytochemicals (Duran and Marcato, 2013). During the past decade, this “controlled release” nanotechnology has gained increasing attention which has been summarized in Table 3 including the previous studies that have successfully encapsulated neem functional ingredients to increase their efficacy. Neem active ingredients predominantly, Azadirachtin can be loaded to both organic nanoparticles (Feng and Peng, 2012) as well as inorganic nanoparticles (Choudhury et al., 2016). Neem leaves comprise of reducing phytochemicals which can be used for the biosynthesis of silver NPs (Shankar et al., 2004). Such NPs capped with neem leaf extracts can act as excellent biopesticide delivery tools for efficient insecticidal activity. Furthermore, neem oil can be loaded onto silica based NPs. These preparations in a study demonstrated significant reduction in *Tuta absoluta*, a tomato leafminer. They presented no significant difference in their insecticidal efficacy when compared with a chemical pesticide, imidacloprid (El-Samahy et al., 2014). In another study, nanoemulsions of neem oil extracted from the seeds of the plant was developed in order to retard the high degradability of neem based biopesticides. A significant reduction of the storage pest *Zabrotes subfasciatus* validated the efficacy of nanoscale carriers in providing stability to the biopesticidal ingredient along with providing controlled release. Such nanoemulsion preparation also presented high UV stability (da costa et al., 2014). On the other hand, loading of neem oil on polymeric nanocarriers, Poly (ε-caprolactone) (PCL) and β-cyclodextrin to control *Bemisia tabaci*, although indicated effective in causing insecticidal activity the efficacy observed was less when compared to the commercial neem oil (Carvalho et al., 2012). Neem seed cake offers potential efficacy as a nano-biofertilizer. Preparation of slow releasing nanostructures containing neem cake stimulates the germination of rhizobacteria along with delivering nutrients to plant (Celsia and Mala, 2014; Mala et al., 2016). Nevertheless, the numerous benefits of NPs in agrochemical delivery have paved way to a new era of biopesticides. This technology provides several benefits including slow release characteristics, enhanced stability of functional ingredients, use of small dose, limited loss by degradation and leaching, ease in handling, transportation and in masking of odor.

**Table 3:** **List of nanocarriers used to encapsulate neem active components and their potential agricultural applications**.

**Neem component**	**Active ingredient**	**Carrier**	**Nanoparticle size**	**Potential application**	**Reference**
Neem	Azadirachtin	Carboxymethyl chitosan with ricinoleic acid (R-CM-chitosan)	200–500 nm	Botanical pesticide	Feng and Peng, 2012
Neem seed kernels	Azadirachtin	Nanoemulsion	1–5 μm	Efficient as a pesticide causing high mortality against a storage pest *Zabrotes subfasciatus*	da costa et al., 2014
Neem oil	Azadirachtin	β-cyclodextrin and PCL	PCL: 4 μm β-cyclodextrin: 83.2 nm	Exhibits high efficacy against nymphs and eggs of *Bemisia tabaci* infecting soybean	Carvalho et al., 2012
Neem seed kernel: -Neem extract -Neem oil	Azadirachtin	PCL	230–245 nm	Exhibits 100% larval mortality against *Plutella xylostella*	Forim et al., 2013
Neem oil	Azadirachtin	Silica NPs	20 nm	Exhibits significant insecticidal effect against *Tuta absoluta*	El-Samahy et al., 2014
Neem leaves	Azadirachtin	Silver NPs	100 nm	Neem coated silver nanoparticles exhibited strong anti-fungal properties (against *Aspergillus terreus*)	Choudhury et al., 2016

## Future perspective

Recently emerging issues regarding the increasing prevalence of pest resistance has prompted the adoption of alternative strategies with special emphasis on integrated pest managements. Neem is an ideal alternative candidate as a natural non-synthetic plant pesticide. Over the years, numerous research has validated its pesticidal activity. It is a cost effective and eco-friendly alternative to the commercial chemically synthesized pesticides. However, owing to its instability to ultraviolet light and limitation of less efficiency as compared to its synthetic counterparts (Barnby et al., 1989), it is vital to develop a novel and efficient strategy to replace toxic chemically synthesized pesticides. This can be achieved by utilizing the past knowledge of neem phytocomponents with pesticidal activity and integrating it with current innovative strategies to develop a unique and effective pest management tool. Amalgamation of nanoscience incorporating organic nanocapsules to provide a dual benefit of controlled delivery of the functional ingredients as well as biodegradable and non-toxic carriers can act as a turning point of modern agriculture. In line with this, inorganic nanoparticles, due to their small size and ease in surface modifications (Joany et al., 2015) can also support the upcoming sustainable agricultural practices. Addition of such nanoformulations can not only act as anti-feedant, ovicidal, sterilant, and morphological and physiological defects in insects but also as a herbal fertilizer. The property of slow release of active ingredients in the soil, conditions the soil and provides nutrients that promote the growth of plants (Mala et al., 2016), which can revolutionize the industry of botanical fertilizers. However, integration of a targeted approach to prevent the side-effects on non-target and ecologically important organisms is an important aspect which still needs to be addressed.

Additionally, this meliaceae plant has unique agro-medicinal properties. Since, parallel to its efficacy as a bio-pesticide it also instills immunomodulatory (Goswami et al., 2016), anti-cancer (Sironmani, 2016), anti-microbial (Verma and Mehata, 2016), and wound healing (Bhagavathy and Kancharla, 2016) properties, which can pave way to an interdisciplinary approach by integrating the attributes of this plant to provide multiple benefits in agriculture as well as in biomedicine.

## Conclusions

The environmental risks associated with the continuous use of synthetic pesticides have prompted the use of plant based insecticidal components that provide selective toxicity to insects with minimum off target effects. The use of botanical pesticides offers eco-friendly pest control strategy to aid the agricultural practices. Among the various herbs, neem plant based insecticides has been the most accepted bio-pesticides, due to the presence of multiple limonoids in neem plant extracts and oil that not only provides a sustainable pest control mechanism but also prevents plant disease resistance, from various synthetic insecticides. Additionally, the efficacy of these pesticidal ingredients of neem can be augmented by encapsulating them in nanocarriers that facilitates in providing sustained and control release of phytochemicals along with site targeted delivery thus, increasing the productivity and yield of crops.

## Author contributions

The primary manuscript was written by SC and JK. Substantial comments and editing was provided by RK, AS, DC, CB, RS, and JK to provide an improved draft. All authors have read and approved the manuscript for publication.

## Funding

The authors would like to thank the Australia-India Strategic Research Fund (AISRF), National Health and Medical Research Council (NHMRC) and Indian Council of Agricultural Research (ICAR) Ph.D. fellowship ID-29-1/2009-EQR/Edn (Pt.III), for financial support.

### Conflict of interest statement

The authors declare that the research was conducted in the absence of any commercial or financial relationships that could be construed as a potential conflict of interest.

## Abbreviations

UNEP: United Nations Environment Programme
WHO: World Health Organization
NLGP: Neem leaf glycoprotein
EC: Ehrlich carcinoma
CD: Cluster of differentiation
NK: Natural killer
IFNγ: Interferon gamma
TNFα: Tumor necrosis factor alpha
SarAg: Sarcoma antigen
PCL: Poly (ε-caprolactone)
DEG: Differentially expressed genes.
